# 1426. Characterization of Patients with Iatrogenic Mycobacterial Infections: A Hospital-Based Survey

**DOI:** 10.1093/ofid/ofac492.1255

**Published:** 2022-12-15

**Authors:** Debdeep Mitra

**Affiliations:** Command Hospital Air Force Bangalore, Bangalore, Karnataka, India

## Abstract

**Background:**

Tuberculosis (TB) is a chronic granulomatous inflammation usually involving the lung parenchyma and hilar lymph nodes. Extra-pulmonary involvement is seen in ∼20% of all TB cases. Developing tuberculosis following treatment for another primary medical condition is a rare occurrence. Iatrogenic literally means illness caused by medical examination or treatment.

Mycobacteria have been used to treat a few medical conditions and the therapeutic use has been validated extensively in literature. The mycobacteria used for therapeutic purposes are supposed to be either attenuated or non pathogenic strains. Growth and dissemination of this mycobacterium is a rare but serious possibility. Reactivation of latent mycobacterial infection following therapy is also a part of iatrogenic mycobacterial infection.

**Methods:**

We report a retrospective study investigating adverse events manifesting with development of iatrogenic tuberculosis. The data was obtained from a tertiary care centre over a period of 2 years. Diagnosis of tuberculosis was established based on clinical, radiological(chest X-Ray and High resolution CT scan), sputum smear microscopy and skin/tissue biopsy and Xpert MTB/RIF. Tuberculin skin test(TST) and Interferon gamma release assay (IGRA) were used as additional diagnostic tests.

**Results:**

The search yielded 26 cases of iatrogenic tuberculosis, with a median age of 56.5 years. Most common cause was reactivation of latent tuberculosis due to use of anti-TNF alpha biologic agents(53.8%). Development of Tuberculosis verrucosa cutis following BCG inoculation for verruca vulgaris was noted in about 19.2% of cases and Tubercular abscess due to Mycobacterium w or BCG inoculation for Covid-19 was noted in 11.5% cases. Bacillus Calmette Guérin(BCG) induced balanitis secondary to therapy for Carcinoma urinary bladder was noted in 7.69%of cases.

**Figure:**
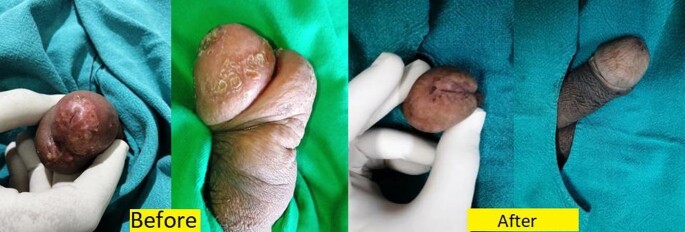

Bacillus Calmette-Guerin or BCG induced balanitis following use of intravesical immunotherapy for treating early-stage urinary bladder cancer and successful clearance of lesions post therapy

**Figure:**
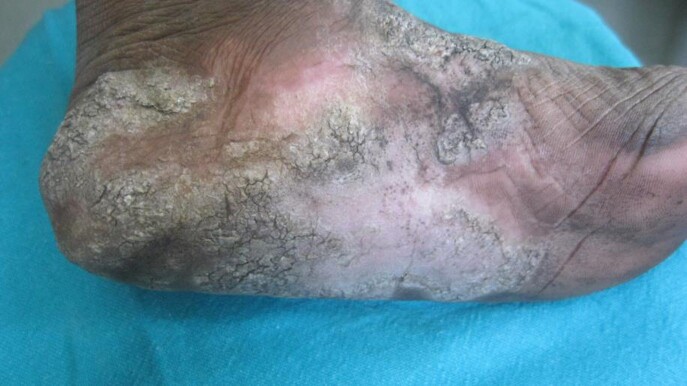

Tuberculosis Verrucosa Cutis following Immunotherapy for plantar warts

**Conclusion:**

All patients were managed successfully with anti tubercular therapy. These observations indicate that tuberculosis infection can develop in previously healthy individuals in endemic zones following biologic therapy of use of mycobacterial agents for other therapeutic indications.

**Disclosures:**

**All Authors**: No reported disclosures.

